# Essential content for teaching implementation practice in healthcare: a mixed-methods study of teams offering capacity-building initiatives

**DOI:** 10.1186/s43058-023-00525-0

**Published:** 2023-11-27

**Authors:** Jessica Reszel, Olivia Daub, Jenny Leese, Hanna Augustsson, Danielle Moeske Bellows, Christine E. Cassidy, Beth E. Crowner, Sandra I. Dunn, Lisa B. Goodwin, Alison M. Hoens, Sarah C. Hunter, Elizabeth A. Lynch, Jennifer L. Moore, Miriam R. Rafferty, Wendy Romney, Dawn Stacey, Implementation Practice CBI Study Team, Sally Bennett, Agnes T. Black, Ashley E. Cameron, Rachel Davis, Shauna Kingsnorth, Julia E. Moore, Christine Provvidenza, Sharon E. Straus, Ashleigh Townley, Ian D. Graham

**Affiliations:** 1 School of Nursing, University of Ottawa, 200 Lees Avenue, Ottawa, ON K1N 6N5 Canada; 2 Centre for Implementation Research, Ottawa Hospital Research Institute, Ottawa, Canada; 3 Better Outcomes Registry & Network (BORN) Ontario, Ottawa, Canada; 4 School of Communication Sciences and Disorders, Western University, London, Canada; 5 School of Epidemiology and Public Health, University of Ottawa, Ottawa, Canada; 6 Procome Research Group, Department of Learning, Informatics, Management and Ethics, Medical Management Centre, Karolinska Institutet, Stockholm, Sweden; 7 Unit for Implementation and Evaluation, Center for Epidemiology and Community Medicine (CES), Stockholm, Sweden; 8 School of Physical Therapy, Massachusetts College of Pharmacy and Health Sciences, Worcester, USA; 9 School of Nursing, Dalhousie University, Halifax, Canada; 10 IWK Health Centre, Halifax, Canada; 11 Physical Therapy, Washington University, St. Louis, USA; 12 Inpatient Rehabilitation, University of Vermont Medical Center, Colchester, USA; 13 Department of Physical Therapy, University of British Columbia, Vancouver, Canada; 14 College of Nursing and Health Sciences, Caring Futures Institute, Flinders University, Adelaide, Australia; 15 Regional Rehabilitation Knowledge Center, Sunnaas Hospital, Oslo, Norway; 16 Institute for Knowledge Translation, Carmel, Indiana, USA; 17 Shirley Ryan AbilityLab and Department of Physical Medicine & Rehabilitation, Northwestern University, Chicago, USA; 18 Physical Therapy, Sacred Heart University, Fairfield, USA

**Keywords:** Implementation practice, Capacity-building initiatives, Mixed-methods, Training content and curriculum

## Abstract

**Background:**

Applying the knowledge gained through implementation science can support the uptake of research evidence into practice; however, those doing and supporting implementation (implementation practitioners) may face barriers to applying implementation science in their work. One strategy to enhance individuals’ and teams’ ability to apply implementation science in practice is through training and professional development opportunities (capacity-building initiatives). Although there is an increasing demand for and offerings of implementation practice capacity-building initiatives, there is no universal agreement on what content should be included. In this study we aimed to explore what capacity-building developers and deliverers identify as essential training content for teaching implementation practice.

**Methods:**

We conducted a convergent mixed-methods study with participants who had developed and/or delivered a capacity-building initiative focused on teaching implementation practice. Participants completed an online questionnaire to provide details on their capacity-building initiatives; took part in an interview or focus group to explore their questionnaire responses in depth; and offered course materials for review. We analyzed a subset of data that focused on the capacity-building initiatives’ content and curriculum. We used descriptive statistics for quantitative data and conventional content analysis for qualitative data, with the data sets merged during the analytic phase. We presented frequency counts for each category to highlight commonalities and differences across capacity-building initiatives.

**Results:**

Thirty-three individuals representing 20 capacity-building initiatives participated. Study participants identified several core content areas included in their capacity-building initiatives: (1) taking a process approach to implementation; (2) identifying and applying implementation theories, models, frameworks, and approaches; (3) learning implementation steps and skills; (4) developing relational skills. In addition, study participants described offering applied and pragmatic content (e.g., tools and resources), and tailoring and evolving the capacity-building initiative content to address emerging trends in implementation science. Study participants highlighted some challenges learners face when acquiring and applying implementation practice knowledge and skills.

**Conclusions:**

This study synthesized what experienced capacity-building initiative developers and deliverers identify as essential content for teaching implementation practice. These findings can inform the development, refinement, and delivery of capacity-building initiatives, as well as future research directions, to enhance the translation of implementation science into practice.

**Supplementary Information:**

The online version contains supplementary material available at 10.1186/s43058-023-00525-0.

Contributions to the literature

- Implementation science knowledge has not been well translated into practice-based settings. Capacity-building initiatives are one way to equip implementation practitioners with the knowledge and skills to apply implementation science in practice.
- We learned from the experiential knowledge of capacity-building initiative developers and deliverers what content on implementation science knowledge and skills is essential to teach practitioners how to implement evidence-informed practices.
- This paper provides a comprehensive description of the content included in past and current implementation practice capacity-building initiatives, which may be used to inform the development and evaluation of future training initiatives.

## Background

With significant time lags between evidence production and implementation [1], there is a long-standing need to accelerate the uptake of research findings into practice to improve healthcare processes and outcomes. The growing implementation science literature provides information on effective methods for moving evidence into practice; however, this scientific knowledge is large, complex, and may be challenging to apply. This has led to a paradoxical research–practice gap, whereby the evidence produced in implementation science is not being applied in real-world practice settings [2]. Thus, there have been recent calls to improve the mobilization of implementation science knowledge beyond the scientific community and into practice settings [3, 4].

Moving implementation science into practice requires a workforce of implementation practitioners who understand how to apply the science of implementation. In this paper, we define “implementation practitioners” as those who are “doing” the implementation of evidence-informed practices, as well as those who are supporting or facilitating implementation efforts [5]. This may include point-of-care staff, managers, quality improvement professionals, intermediaries, implementation support staff, and policymakers. To build the workforce of implementation practitioners, there is a need for training and professional development opportunities, which we call “capacity-building initiatives.” While there are an increasing number of implementation capacity-building initiatives available [6, 7], these programs often focus on teaching researchers about implementation science, with fewer aimed at teaching how to apply implementation science to improve implementation of evidence in practice settings (i.e., implementation practice) [7–9]. A recent systematic review [7] of the academic literature included 31 papers (reporting on 41 capacity-building initiatives) published between 2006 and 2019. The review found that many capacity-building initiatives were intended for researchers at a postgraduate or postdoctoral level, and there were fewer options for implementation practitioners working in practice settings.

While there are some examples of practitioner-focused capacity-building initiatives in the literature [10–22], most are being developed and delivered in isolation and not published in the academic or grey literature. In addition, reviewing this literature revealed that most of these publications focus on reporting evaluations of the short- and long-term outcomes of the capacity-building initiatives with only high-level details of the specific training content and the rationale for this content. Despite the development of competencies [23, 24] and frameworks [22] for implementation research and practice that have been informed through primary studies, literature reviews, and convening experts, to our knowledge, there has not been a consensus-building approach to date. Thus, there is limited synthesized information on what content is currently included in implementation practitioner capacity-building initiatives and no universal agreement or guidance on what content should be included to effectively teach implementation practitioners.

The increasing demand for and offerings of implementation practice capacity-building initiatives provide an opportunity to synthesize and learn from the individuals and teams offering this training. Our research team, which is composed of implementation scientists, implementation practitioners, clinicians, health leaders, and trainees, conducted a mixed-methods study to explore the experiences of teams offering capacity-building initiatives focused on implementation practice to inform the future development of high-quality training initiatives. The study had three aims. The first aim, which is the focus of this paper, was to describe what capacity-building initiative developers and deliverers identified as essential training content for teaching implementation practice. The other two aims (to be reported on elsewhere) were to describe and compare the similarities and differences between the capacity-building initiatives (e.g., structure, participants) and explore the experiences of those developing and delivering capacity-building initiatives for practitioners.

## Methods

We used the Good Reporting of a Mixed Methods Study (GRAMMS) checklist [25] to inform our reporting (Additional file 1).

### Study design

The overall study was a convergent mixed-methods study [26] (cross-sectional survey and qualitative descriptive design [27]) that applied an integrated knowledge translation approach [28] where all study participants were invited to contribute to the analysis, interpretation, and reporting of the study. Here we report on one component of the larger study. Specifically, we focus on a sub-set of the quantitative and qualitative data reporting on the content and curriculum of the capacity-building initiatives.

### Study participants

We enrolled English-speaking individuals who had experience developing and/or delivering a capacity-building initiative that focused on teaching learners how to apply implementation science knowledge and skills to improve the implementation of evidence-informed practices in practice settings. The capacity-building initiatives must have been offered in the last 10 years and could be offered in any geographical location or online. We excluded capacity-building initiatives that focused on training researchers or graduate students to undertake implementation research.

We used purposive sampling. First, using the professional networks of the study team, we compiled a list of capacity-building initiatives and the primary contact (e.g., training lead). Second, three team members (JR, IDG, AM) independently screened the capacity-building initiatives included in Davis and D’Lima’s systematic review [7], consulting the full-text papers as needed to identify initiatives focused on implementation practice. Finally, we used snowball sampling to identify other individuals who had developed and delivered capacity-building initiatives. The first author (JR) invited the potential participants by email. If no response was received, an email reminder was sent 2 weeks and 4 weeks after the initial invitation.

Once the primary contact for a capacity-building initiative was enrolled, they had the opportunity to share the study invitation with their other team members. This resulted in some capacity-building initiatives having more than one person enrolled in the study, providing multiple perspectives on the development and delivery of the initiative. For simplicity, we refer to them as “teams” regardless of whether there was one person enrolled or multiple people enrolled.

### Data collection

First, participants completed an online questionnaire developed by the study team, which included closed-ended and open-ended questions (Additional file 2 presents the sub-set of questions used in this analysis that focused on the content and curriculum). The questionnaire was piloted internally by two team members, and minor changes were made to improve functionality (e.g., branching logic), comprehensiveness (e.g., adding in open text boxes for respondents), and clarity (e.g., defining key terms used). We asked for one completed questionnaire per capacity-building initiative. When there were multiple team members enrolled in the study, they could nominate one person to complete the questionnaire on their behalf or complete the questionnaire together.

After completing the questionnaire, all participants were interviewed individually or in a focus group via videoconference to explore the questionnaire responses and discuss the capacity-building initiative in more detail. Individual interviews were used when there was only one team member enrolled; focus groups were used when there were two or more team members. The interviews and focus groups were facilitated by one of three research team members, all of whom identified as women and were trained in qualitative interviewing: JR is a master’s prepared registered nurse; OD is a master’s prepared speech-language pathologist with doctoral training in health rehabilitation sciences research and a knowledge translation specialist; JL is a doctoral prepared researcher with expertise in patient engagement. A semi-structured question guide was developed by the first and senior author (JR, IDG) and shared with the broader team. We used the team feedback to update the question guide, including adding new questions and probes, re-ordering the questions to improve flow, and refining the wording of the questions for clarity (Additional file 2 presents the sub-set of questions used in this analysis that focused on the content and curriculum).

Finally, we asked participants to share any capacity-building initiative materials to provide further details (e.g., scientific or grey literature publications, website materials, training agendas, promotional materials). We only collected publicly available materials to minimize concerns around the sharing/disclosing of proprietary content.

The questionnaire and publicly available materials provided data on what content is currently included in the capacity-building initiatives. The interview and focus group data provided information on why certain content was included, as well as how and why content changed over time. Together, this provided information on what we have labeled “essential content,” which is a reflection of both what study participants have chosen to include in their training initiatives, and their views on priority content areas for implementation practitioners based on their own experiences developing and delivering the initiatives.

### Data analysis

Closed-ended questionnaire responses were analyzed using descriptive statistics. Frequencies (counts and percentages) were calculated for nominal data. Medians and ranges were calculated for continuous data. The questionnaire responses, qualitative transcripts, and course materials shared by participants were uploaded to NVivo12Pro for data management [29]. The merged dataset was analyzed using conventional content analysis, with the codes emerging inductively from the data [30]. Two authors (JR, OD) started by independently reading the data and coding all segments that pertained to training content and curriculum. They met regularly to compare their coding, discuss and resolve differences, build and revise the coding scheme, and group codes into categories. When the coding scheme was well-developed, and the coders were coding consistently (which occurred after coding data from one-third of the teams), the remaining data were coded by either JR or OD. The coding was then audited by one of seven members of the broader research team (HA, DMB, LBG, AMH, SCH, AEL, DS). These seven team members were “senior reviewers” with subject matter expertise in implementation science and practice [31]. They audited the coding and offered their feedback on how the text segments were labeled and categorized. This feedback was discussed by the two primary coders (JR, OD) and the senior author (IDG). The review process resulted in (1) changes to which codes were applied to specific text segments, (2) changes to the coding structure, including splitting existing codes into more precise labels, and (3) re-organizing existing codes into new categories. The final coding scheme was applied to the data. Finally, we categorized the identified theories, models, frameworks, and approaches (i.e., other methods in implementation) (TMFAs) [32] according to the three main aims described by Nilsen [33]: to guide, to understand or explain, or to evaluate implementation. We also categorized the identified implementation steps and skills according to the three phases in the Implementation Roadmap [34]: issue identification and clarification; build solutions; and implement, evaluate, sustain.

### Integration of quantitative and qualitative data

We used integration approaches at several levels. At the methods level, we used *building* where the interview probes were developed based on questionnaire responses [26]. We also used *merging* by bringing the questionnaire and interview/focus group data together for analysis [26], giving both datasets equal priority. At the interpretation and reporting level, we used a *narrative weaving* approach to describe the categories informed by both datasets [26]. The integration of quantitative and qualitative data contributed to an *expansion* of our understanding of the capacity-building initiative content [26], with the questionnaire contributing to identifying what content is included and the interview/focus group data providing the rationale for the content.

### Strategies to enhance methodological rigor

Dependability and confirmability [35] were enhanced by maintaining a comprehensive audit trail including raw data (e.g., verbatim transcripts), iterations of coding and coding schemes, and notes from data analysis meetings. To enhance credibility and confirmability [35], 35% of the data were coded independently by two people. All study participants were sent a summary of their data prepared by the research team and were asked to review it for accuracy and comprehensiveness (i.e., member checking the data). In addition, having senior reviewers with content expertise audit the coding helped make sense of the different implementation concepts and terms in the data, ensuring that data were coded and categorized accurately. Finally, interested study participants were involved in the sense-making process through their involvement in writing and critically revising this manuscript. We aimed to facilitate an assessment of the transferability [35] of the findings by describing contextual information on the capacity-building initiatives and study participants.

## Results

**Fig. 1:**
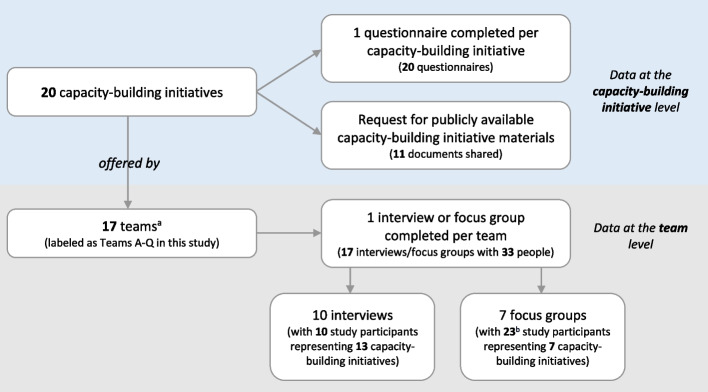
Summary of data collected. ^a^One team reported on 3 capacity-building initiatives and one team reported on 2 capacity-building initiatives. All other teams reported on 1 capacity-building initiative only. Teams participating in this study comprised between 1 and 6 people. ^b^Five study participants took part in two focus groups

We enrolled 33 people (representing 17 teams) who developed and delivered capacity-building initiatives focused on implementation practice. Collectively, these 33 study participants shared information on 20 unique capacity-building initiatives that were offered by their 17 teams (Fig. 1). We indicate the denominator throughout the results to make clear when the results refer to capacity-building initiative level data, which was largely collected through the questionnaire and the shared capacity-building initiative materials (*n* = 20) or team-level data, which was largely collected through interviews and focus groups (*n* = 17).

Between September 2021 and November 2022, we collected 20 questionnaire responses (i.e., one per capacity-building initiative) and conducted 10 online interviews and 7 online focus groups (i.e., one per team) (Fig. 1). The focus groups included between 2 to 6 people. Interviews lasted an average of 60 min (range = 51–77 min) and focus groups lasted an average of 68 min (range = 51–79 min). We received materials for 11 out of 20 capacity-building initiatives, specifically: 6 publications, 2 course agendas, 2 course advertisements, and 1 website.

### Study participants

The 33 study participants represented a blend of both research and practice experience. Half of the study participants (*n* = 17/33, 52%) currently identified as both a research professional (researcher or implementation scientist) and a practice-based professional (clinician or implementation practitioner). Three-quarters of study participants (*n* = 24/33, 73%) were currently involved in implementation in practice settings (clinician or implementation practitioner or manager/leader) (Table 1). Nearly all study participants reported having experience in implementation practice (*n* = 31/33, 94%). Of those with experience, the median number of years’ experience was 9 (range = 4–30 years).

**Table 1:** Demographic characteristics of study participants (*N* = 33)

**Characteristic**	
Gender—*n* (%)	
Woman	27 (82)
Man	6 (18)
Current professional identity—*n* (%)^a^	
Researcher or implementation scientist	26 (79)
Clinician^b^	14 (42)
Implementation practitioner	17 (52)
Manager/leader	3 (9)
Years of experience—median (range)	
Developing capacity-building initiatives	6 (0–22)
Delivering capacity-building initiatives	5 (1–22)
Providing adult education	10 (0–36)

^a^Participants were able to select more than one response option

^b^Study participants had clinical training in disciplines such as kinesiology, medicine, nursing, nutrition, occupational therapy, physical therapy, psychology, public health, and speech-language pathology

### Contextual information on capacity-building initiatives

The capacity-building initiatives (*n* = 20) had been offered a median of 4 times (range = 1–35 offerings) between 2009 and 2022 (Table 2).

**Table 2:** Contextual information on capacity-building initiatives (*N* = 20)

**Characteristic**	
Type of organization offering capacity-building initiative—*n* (%)^a^	
Health services organization	10 (50)
Academic organization	6 (30)
Organization providing implementation support	5 (25)
Professional association	2 (10)
Private sector organization	1 (5)
Mode of delivery of capacity-building initiative—*n* (%)	
Combination of online and in-person	10 (50)
In-person only	6 (30)
Online only	4 (20)
Length of capacity-building initiative (hours)—median (range)^b^	
16 (3–30)	
Location where capacity-building initiative was offered—*n* (%)^c^	
North America	9 (45)
Australia	7 (35)
Europe	3 (15)
Asia	2 (10)
Africa	1 (5)
Offered online only	4 (20)
Types of participants that take part in capacity-building initiative—*n* (%)^a^	
Point-of-care clinicians	19 (95)
Implementation/quality improvement leads	18 (90)
Managers/administrators	17 (85)
Researchers	16 (80)
Intermediaries/implementation support staff	13 (65)
Graduate and postgraduate trainees	13 (65)
Evaluators	8 (40)
Policymakers	7 (35)
Funders	5 (25)
Health consumers or patients	5 (25)
Focus of capacity-building initiative—*n* (%)	
General—not specific to a specific context	13 (65)
Rehabilitation	5 (25)
Dementia	1 (5)
Patient safety	1 (5)
Minimum entry requirements for capacity-building initiative—*n* (%)^a^	
No specific requirements	13 (65)
Specific professional background or discipline	7 (35)
Membership or affiliation with a specific organization or group	3 (15)
Attendance at a specific conference or meeting	2 (10)

^a^Respondents could select more than one response option

^b^Data reflects direct teaching time and does not include additional time learners may spend on self-directed learning or mentorship activities

^c^Some initiatives were offered in more than one location

### Capacity-building initiative content

**Fig. 2:**
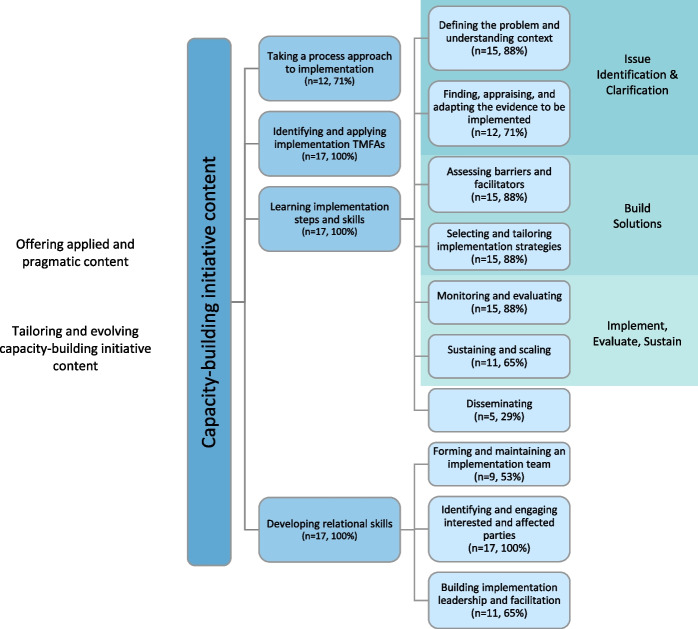
Organization of study findings. The number of teams that discussed each category is indicated in brackets; the teams could have identified/described the category in any or all of the data sources: questionnaire, interview or focus group, shared capacity-building initiative materials. *TMFAs *theories, models, frameworks, approaches

Nine of 17 teams (53%) explicitly described their capacity-building initiatives as introductory level. Study participants identified a variety of content areas included in their capacity-building initiatives, which we present according to four categories and 10 sub-categories, as well as the overarching categories of applied and pragmatic content and tailoring and evolving content (Fig. 2). Illustrative quotes are presented in Table 3.

**Table 3:** Illustrative quotes

Category	Sub-category	Illustrative quotes
1. Taking a process approach to implementation	—	“A lot of people don’t recognize how long this [implementation] process can take…I think helping people understand that this is a dynamic process and that it is fluid and things will change. Flexibility is important and sometimes there will be other competing priorities that this [implementation project] might have to go on hold for, and that’s okay.” (Team I)
2. Identifying and applying implementation TMFAs	—	“They [learners] struggle with many of the frameworks including applying the frameworks and being able to see how this works in their context. How would they apply it? Do they need to adapt it? What does that literally look like?” (Team N)
3. Learning implementation steps and skills	3.1 Defining the problem and understanding context	“Often a course jumps straight into cycles and context and the intervention and the stakeholders and the context. But what we often were finding with a lot of different clinician groups was that the conceptualization of the problem was pretty shaky and the healthcare providers really jumped to solutions. So we have spent quite a lot of time pondering how we get to the bottom of a problem.” (Team D)
	3.2 Finding, appraising, and adapting the evidence to be implemented	“The challenge is that groups think they want to implement something, but they haven’t actually figured out what is the thing that needs to be implemented? And is there good evidence for it? And are we doing the right thing by wanting to implement it? Or is this just ‘we heard about it and we think it’s a good idea,’ but maybe there isn’t really good evidence for it?” (Team H)
	3.3 Assessing barriers and facilitators	“The barriers and facilitators piece is absolutely fundamental because our process historically has just been ‘I want you to adopt this new thing.’ Then I tell you in a meeting and then I hope you just do it. I don’t think about like have I convinced you it’s important? Do you know where to find the form? Is there an algorithm that tells you when and how to do this? Why should you do it? How’s it going to impact patient care? Whatever practice you’re trying to change, what keeps them from changing?” (Team A)
	3.4 Selecting and tailoring implementation strategies	“What are their barriers or facilitators and what strategies will they use to support people to change their behavior? In my mind, if people don’t have that then nothing else matters; there’s no reason to proceed because chances are you arbitrarily picked implementation strategies not knowing they were implementation strategies, and you will move forward with them even though they don’t address underlying barriers to change.” (Team N)
	3.5 Monitoring and evaluating	“Clinicians may or may not appreciate the value of measuring the evidence practice gap at the beginning and then again later on […] It wasn’t just for research purposes, but being able to communicate to the team that what you’ve been doing has made this amount of change.” (Team L) “They [learners] are often people who haven’t had much experience with measurement and evaluation and often feel a bit threatened by measurement and evaluation. So it’s really trying to think about what might be simple things that you could measure? What are the meaningful things? How does it relate to your problem?” (Team D)
	3.6 Sustaining and scaling	“When we were getting close to wrapping up, we look at the [Knowledge-to-Action] cycle again and outcomes of interest—what metrics they wanted to measure and issues around sustainability, explaining how difficult it is to sustain these efforts in many settings.” (Team K)
	3.7 Disseminating	“It’s important to provide a fulsome understanding of what knowledge translation encompasses. So looking at some of the jargon or buzz words that people hear and where they sit within the knowledge translation umbrella of dissemination and implementation and really helping people to understand the difference between the two.” (Team J)
4. Developing relational skills	4.1 Forming and maintaining an implementation team	“We cover how to assemble the right team to develop and progress a project to support implementation of a specific innovation.” (Team G)
	4.2 Identifying and engaging interested and affected parties	“Part of the training was learning to engage them [clinical team members and administrators] early in the process and knowing that they had to do that rather than just going in and doing what they wanted to implement and then saying ‘oh we’re doing this by the way.’” (Team L)
	4.3 Building implementation leadership and facilitation	“A decent amount of time in our program is devoted to leadership development—thinking about yourself as a leader and thinking about facilitation and relationships…those are skills not everybody has, so people need ongoing development in those areas.” (Team O)
6. Offering applied and pragmatic content	—	“It [capacity-building initiative] was an opportunity to bring people together to expose them to an array of tools. The feedback that we got from people was that they were so grateful that somebody had done this compilation for them and that they now had a package that they could walk away with and use in the way that they felt most appropriate.” (Team B)
7. Tailoring and evolving capacity-building initiative content	—	“We’re constantly tweaking and adjusting the content to tailor it to the personalities and the projects. It is a very comprehensive full on three-days, but that’s just our personalities to want to make it really tailored…we don’t just rinse and repeat the same course because it’s easy.” (Team F) “One of the biggest things that we’ve done in the last couple of years is really making sure that we take an intersectionality lens right from the very beginning and get people to reflect on our positions within the project, the biases that we bring, and thinking about who is on the team? Who is not on the team?” (Team P)

*TMFAs* Theories, models, frameworks, approaches

#### Taking a process approach to implementation

Twelve teams (*n* = 12/17, 71%) described the importance of teaching learners to take a process approach to implementation. Participants highlighted that because learners tend to be action-focused, they needed to include content on the importance of taking a thoughtful approach and not jumping in too quickly without a thorough plan. To do this, these teams included content on how to develop a comprehensive implementation plan. Teaching this process approach also required information on how long the process can take, its iterative nature, and the need to be adaptable as things change.

#### Identifying and applying implementation TMFAs

All 17 teams reported that their capacity-building initiatives included two or more implementation TMFAs. In total, study participants identified 37 unique TMFAs that were introduced in their capacity-building initiatives (Table 4). The most common were the Knowledge-to-Action Framework (*n* = 14/20), Theoretical Domains Framework (*n* = 11/20), COM-B Model for Behavior Change (*n* = 9/20), RE-AIM (*n* = 9/20), Consolidated Framework for Implementation Research (*n* = 8/20), and the Behavior Change Wheel (*n* = 5/20). The remaining TMFAs were all used by four or fewer capacity-building initiatives.

**Table 4:** Theories, models, frameworks, and approaches (*n* = 37) included in the 20 capacity-building initiatives

**Theories, models, frameworks, and approaches (TMFAs)**^a^	**Number of capacity-building initiatives that included TMFA**
KTA (Knowledge-to-Action) Framework [36]	14
TDF (Theoretical Domains Framework) [37]	11
COM-B Model for Behavior Change [38]	9
RE-AIM (Reach, Effectiveness, Adoption, Implementation, and Maintenance) [39, 40]	9
CFIR (Consolidated Framework for Implementation Research) [41, 42]	8
Behavior Change Wheel [38]	5
Dynamic Sustainability Framework [43]	4
NHS (National Health Service) Sustainability Model [44]	4
Interactive Systems Framework for Dissemination and Implementation [45]	3
PARiHS (Promoting Action on Research Implementation in Health Services) Framework [46]	3
EBSIS (Evidence-based System for Innovation Support) [47]	2
Integrated Knowledge Translation [48]	2
NPT (Normalization Process Theory) [49, 50]	2
Proctor’s Implementation Outcomes [51]	2
Quality Implementation Framework [52]	2
Rogers Diffusion of Innovation [53]	2
ADAPT Process Model [54]	1
Awareness-to-Adherence Model [55]	1
CAN-Implement [56]	1
Complexity theory [57]	1
End-of-Grant Knowledge Translation [48]	1
EPIS Framework (Exploration, Preparation, Implementation, Sustainment) [58, 59]	1
Forms and Functions [60]	1
FRAME (Framework for Reporting Adaptations and Modifications-Expanded) [61]	1
IHI (Institute for Healthcare Improvement) Model for Improvement [62]	1
IHI (Institute for Healthcare Improvement) Psychology of Change Framework [63]	1
Implementation Process Model [64]	1
Iowa Model of Evidence-Based Practice [65]	1
Network Mapping [66, 67]	1
OMRU (Ottawa Model of Research Use) [68]	1
R = MC2 Readiness Framework [69]	1
Soft Systems Theory [70]	1
Systems Thinking [71]	1
Systems Pathways [57]	1
Systems Readiness [72]	1
TACT-A (Target, Action, Context, Timing, Actors) Framework [73]	1
Theory of Planned Behavior [74]	1

^a^Presented in order of frequency

Eleven capacity-building initiatives used a TMFA as the underpinning structure for the training content: nine were based on the Knowledge-to-Action framework, one was based on the Behavior Change Wheel, and one was based on the Awareness-to-Adherence Model.

Of the 20 capacity-building initiatives, 16 (80%) included at least one TMFA that *guides* implementation, 16 (80%) included at least one TMFA that *explains* implementation, and 10 (50%) included at least one TMFA to *evaluate* implementation. Eight of the 20 capacity-building initiatives (40%) included TMFAs from all three aims; six (30%) included TMFAs from two aims (guide/explain = 4, explain/evaluate = 1, guide/evaluate = 1); and six (30%) included TMFAs from one aim only (guide = 3, explain = 3).

Nine teams (*n* = 9/17, 53%) described the importance of focusing on the “how,” showing learners the menu of options and helping them to understand how to appropriately select and apply TMFAs to the different stages of their implementation projects. One team described introducing tools to facilitate the selection of TMFAs (e.g., Dissemination & Implementation Models in Health [75], T-CaST [76, 77]).

Teams noted that the content on TMFAs was often challenging for learners, with one team describing it as “bamboozling” (Case M). Challenges were due to learner anxiety with the academic nature and language of TMFAs, as well as difficulties understanding how they can be applied to their work. To address these challenges, teams changed their capacity-building initiative content to make it less theoretical (i.e., less focus on *telling* them about theories), with an increased focus on how to apply theory in implementation projects. Other teams described including information to reinforce the flexible application of TMFAs, emphasizing the ability to try one out and re-visit the choice if it is not meeting the project needs.

#### Learning implementation steps and skills

All 17 teams described how their training content focused on practical implementation skills to complete various steps in the process. Teams described seven core steps (Fig. 2).

##### Defining the problem and understanding context

Fifteen teams (*n* = 15/17, 88%) identified the importance of teaching learners to clearly define what problem the implementation project is aiming to address. Examples of this content included: clarifying what the problem is, understanding the context and current practice, using data to show the problem (evidence-practice gap), understanding the root cause of a problem, defining a problem that is specific and feasible to address, and understanding the problem from different perspectives.

Teams described spending a significant amount of time on this content due to its foundational nature for learning about subsequent steps in the implementation process. However, one participant cautioned the need to strike a balance between helping learners to thoroughly define and understand their problem without going so in-depth that they lose sight of what they are trying to accomplish within their implementation project.

##### Finding, appraising, and adapting evidence

Many teams (*n* = 12/17, 71%) described content about the evidence to be implemented as critical, including how to find, appraise, and adapt evidence for the context in which it is being implemented. Several teams described how learners could be quick to select the evidence to be implemented based on hearing about something “bright and shiny” (Team M), learnings from conferences and meetings, or papers reporting on a single study. Because of this, training content on how to conduct a more comprehensive search and appraisal of the evidence was essential.

Specifically, teams included content on the importance of ensuring there is evidence to support what is being implemented, how to search for research evidence, the importance of considering other forms of evidence such as staff and patient experiences, how to merge research evidence with experiential knowledge, considerations for ensuring the fit of the evidence to be implemented with the implementation setting, and understanding the appraised quality and levels of evidence (e.g., the evidence pyramid). Two teams (*n* = 2/17, 12%) acknowledged that even after learners acquired some knowledge and skills to search for and appraise evidence, they rarely had the time to undertake these tasks in their day-to-day professional roles. Therefore, making learners aware of resources to support this work was important.

Seven teams (*n* = 7/17, 41%) described training content related to adapting the evidence, practice, or innovation being implemented to fit with the local context. The concept of adaptation could be challenging for learners accustomed to working in a more “top-down” or directive model, where they assumed the evidence, practice, or innovation would be implemented as is. In these cases, teams identified that it was especially important to include information on how the organizational context and group needs should be considered to optimize the uptake and sustainability of the evidence, practice, or innovation being implemented.

##### Assessing barriers and facilitators

Fifteen teams (*n* = 15/17, 88%) discussed the fundamental importance of including content on how to systematically assess for barriers and facilitators that are likely to influence implementation. Teams shared how learners may either skip right from evidence selection to implementation solutions or erroneously believe that simply telling people a change is being made should be enough to result in behavior change. Teaching learners about the determinants that may influence the adoption (or lack of adoption) of evidence and the process for identifying these determinants was, therefore, identified as critical by nearly all teams. The content for this stage frequently included different TMFAs to guide the work (e.g., Theoretical Domains Framework [TDF] [37], Consolidated Framework for Implementation Research [CFIR] [41, 42]).

##### Selecting and tailoring implementation strategies

Fifteen teams (*n* = 15/17, 88%) highlighted the importance of teaching learners how to select implementation strategies using a structured approach that aligns with and addresses the identified barriers. Teams shared that learners may default to using familiar implementation strategies (such as education); therefore, teaching about the full range of implementation strategies was important. The capacity-building initiatives frequently included content and activities on how to map identified barriers to specific evidence-based implementation strategies and how to prioritize which ones to select. Again, teams described relevant resources (such as the Expert Recommendations for Implementing Change [ERIC] Taxonomy [78], the Behavior Change Technique [BCT) Taxonomy [79], and the Behavior Change Wheel [38]) that they used to help learners understand and apply the implementation strategy selection process.

##### Monitoring and evaluating

Fifteen teams (*n* = 15/17, 88%) described training content related to monitoring and evaluating implementation. Teams shared how they reinforced the importance of evaluating implementation projects to make course corrections and show the impact of their work. Three teams (*n* = 3/17, 18%) acknowledged that monitoring and evaluation can be unfamiliar and intimidating to learners and ensured that the content covered the “nuts and bolts” of monitoring the implementation process and conducting an outcome evaluation. Five capacity-building initiatives (*n* = 5/20, 25%) included logic models as a tool to plan for evaluations; other TMFAs included RE-AIM [39, 40] and Proctor’s implementation outcomes [51].

##### Sustaining and scaling

Eleven teams (*n* = 11/17, 65%) stated they included content on sustainability, such as tools for sustainability planning, determinants of sustainability, strategies for assessing and enhancing sustainability, and challenges with sustaining change over time. One team (*n* = 1/17, 6%) described including information on spread and scale. Although this content was often introduced near the end of the capacity-building initiative, teams reminded learners that sustainability needs to be considered at the beginning and throughout the implementation process.

##### Disseminating

Five teams (*n* = 5/17, 29%) included content on how to disseminate the findings of implementation projects. Content included strategies to disseminate project findings to interested and affected parties and decision-makers, as well as dissemination through scientific venues such as conference presentations and publications.

While all capacity-building initiatives (*n* = 20/20) focused on the implementation of evidence into practice, two teams (*n* = 2/17, 12%) also included information on how to undertake a dissemination project (e.g., developing a resource to share evidence). Teams also described the need to teach learners about the full spectrum of knowledge translation and the distinction between dissemination and implementation.

#### Developing relational skills

All teams (*n* = 17/17) discussed the importance of learning about the relational skills required throughout the implementation process, with one participant describing it as the “most neglected part of capacity building” (Case N).

Teams identified three main content areas for teaching these relational skills: forming and maintaining an implementation team, identifying and engaging interested and affected parties, and building implementation leadership and facilitation. Cutting across these three main areas, there were general examples of other relational content, including how to build trusting relationships, work inter-professionally, navigate power differences and hierarchies, and communication skills.

##### Forming and maintaining an implementation team

Nine teams (*n* = 9/17, 53%) discussed content on how to build an implementation team and define roles, how to manage team dynamics, and how to engage members throughout the implementation project.

##### Identifying and engaging interested and affected parties

All teams (*n* = 17/17) described content related to identifying and engaging interested and affected parties. Topics included the value of engagement, identifying and mapping key influencers, strategies for engagement, tailoring engagement approaches, and evaluating engagement.

Fourteen teams (*n* = 14/17, 82%) stated they included content on the importance of engaging health consumers (e.g., patients, families, caregivers). While some capacity-building initiatives only briefly discussed this, others described more detailed content, such as the rationale for and importance of consumer engagement, guidance for reimbursing health consumer partners, and strategies for working with health consumers. Two teams (*n* = 2/17, 12%) highlighted the importance of having this content delivered by health consumers themselves to showcase their experiences and stories.

##### Building implementation leadership and facilitation

Eleven teams (*n* = 11/17, 65%) included content on the knowledge and skills needed to be a facilitator of the implementation process including: the role of the facilitator, effective leadership, change management, managing resistance, and motivating others. Learners entering the capacity-building initiative may not recognize their ability to be an implementation leader; it was, therefore, important to include content that encouraged learners to reflect on their current attitudes and skills as a leader, work on leadership development, and help learners see themselves as leaders of implementation.

#### Offering applied and pragmatic content

All teams (*n* = 17/17) discussed the importance of applied content for teaching implementation practice. Teams acknowledged the growing and complex implementation science literature and highlighted the importance of content that effectively distills this literature into pragmatic and accessible content for learners (e.g., top five tips, toolkits, case examples). Teams reported that including practical tools and resources in the capacity-building initiatives was important so that learners had something tangible they could apply in their practice. Thirteen teams (*n* = 13/17, 76%) named at least one additional resource that they shared with learners. Twenty-seven unique resources were identified (Table 5).

**Table 5:** List of additional resources (*n* = 27) shared in the 20 capacity-building initiatives

**Additional resources (*n* = 27) shared in the capacity-building initiatives (presented in alphabetical order)**
APRAISE Tool [80]
Behavior Change Technique (BCT) Taxonomy [79]
CADTH Rx for Change Database [81]
Methods of Patient & Public Engagement: A Guide [82]
CIHR’s A Guide to Evaluation in Health Research [83]
Dissemination & Implementation Models in Research & Practice [75]
Eisenhower Matrix [84]
EPOC (Effective Practice and Organisation of Care) Taxonomy [85]
ERIC (Expert Recommendations for Implementing Change) Taxonomy [78]
Getting to Outcomes [86]
Health Consumers Queensland [87]
IAP2 (International Association for Public Participation) [88]
Institute of Health Economics report on Effective Dissemination of Research Findings [89]
Implementation Mapping [90]
King’s Improvement Science (KIS) Guide to Evaluation Resources [91]
Knowledge Translation Planning Template [92]
KTDRR (Center on Knowledge Translation for Disability & Rehabilitation Research) KT Strategies Database [93]
NHS (National Health Service) Institute for Innovation and Improvement Sustainability Guide [94]
NIRN (National Implementation Research Network) [95]
Ready, Set, Change! Decision Support Tool [96]
SBAR Tool (Situation, Background, Assessment, Recommendation) [97]
Sustain Tool (PSAT [Program Sustainability Assessment Tool] and CSAT [Clinical Sustainability Assessment Tool]) [98]
T-CAST (Theory, Model, and Framework Comparison and Selection Tool) [76, 77]
The 7 Ps (Programs, Practices, Policies, Procedures, Principles, Pills, Products) [99]
The Engagement Toolkit [100]
Theory and Techniques Tool [101]
TICD (Tailored Implementation for Chronic Diseases) Checklist [102]

#### Tailoring and evolving capacity-building initiative content

Seven teams (*n* = 7/17, 41%) described the importance of tailoring the content to each group of learners. While some teams acknowledged that there is content that is “locked in” or “universal,” other content can be tailored to meet the specific needs of learners (for example, based on learners’ area of practice, implementation projects, baseline knowledge, and learning needs).

Of the 20 capacity-building initiatives, 17 (85%) had been offered more than one time. These teams described changes to their training content over time (Table 6). These content changes were prompted by feedback received via formal learner evaluation forms; informal check-ins with learners during the capacity-building initiative; observations of what learners are asking questions about or struggling with; and new developments in the fields of knowledge translation, implementation science, and adult education.

**Table 6:** Examples of changes to content in capacity-building initiatives

**Type of change to capacity-building initiative content**	**Examples**
Removing content or decreasing content that was not aligned with learners’ needs or preferences	• Removing specific tools and resources that were not helpful to learners • Decreasing theoretical content • Removing content that was not relevant to learners (e.g., media training)
Adding content or increasing time spent on content that aligns with learners’ needs or preferences	• New developments in implementation science • Specific implementation steps such as clarifying the problem, consumer engagement • Adding new tools and resources developed by the capacity-building initiative team (e.g., implementation decision aids, workbooks)
Changing the presentation of content	• Reducing the depth of content • Shifting to more practical content • Increasing the number of case examples • Updating the language used to align with developments in the field

Teams shared emerging topics that are becoming increasingly important to include in their capacity-building initiatives. More recent offerings of the capacity-building initiatives have taught learners about taking an intersectionality lens, considerations for equity, diversity, and inclusion, and applying a principled approach to partnerships.

## Discussion

This study aimed to describe what capacity-building initiative developers and deliverers identify as essential content for teaching implementation practice. Based on the experiences of 17 teams that delivered 20 capacity-building initiatives, we identified four categories of content including taking a process approach to implementation, implementation TMFAs, implementation steps and skills, and relational skills, as well as the overarching categories of applied and pragmatic content, and tailored and evolving content. These findings provide an overview of the content being covered by a variety of capacity-building initiatives worldwide and the rationale for this content. Learning about the rationale for the content provided insights into some of the challenges current and aspiring implementation practitioners face both in the learning process and in their practice settings. These findings provide a foundation for building, refining, and researching capacity-building initiatives to further develop the implementation practice workforce, which is essential for scaling the implementation of evidence globally.

In this study, teams identified 37 different TMFAs and 27 additional resources that were introduced across the 20 capacity-building initiatives. While some of these were applied across a substantial number of capacity-building initiatives (e.g., Knowledge-to-Action framework [36]), most were used infrequently. This finding signals a general lack of consensus about what TMFAs and resources to use, a finding reported elsewhere [103]. A recent scoping review identified over 200 knowledge translation practice tools (i.e., tools that guide how to do knowledge translation) [104]. This has created a potentially overwhelming number of TMFAs that are used infrequently and/or inappropriately [105, 106], with many practitioners reporting a lack of confidence in choosing a framework [107]. It is worth reflecting on whether the people developing and delivering capacity-building initiatives are propagating this challenge by sharing and endorsing so many TMFAs and resources, especially without equipping learners with the tools needed to select and implement appropriate TMFAs. While some teams in our study did describe the importance of content on how to identify and select appropriate TMFAs, only one team identified the use of selection tools to facilitate this process. As more practice-based selection tools are developed and tested [104, 106], they may be helpful to implementation practitioners as they explore the large number of potential TMFAs to apply in their work.

The capacity-building initiatives in this study aligned with current understanding of core pillars [22] and essential competencies for implementation practice [23, 108]. Leppin and colleagues [22] identified three core pillars: understanding evidence-based interventions and implementation strategies; using theories, models, and frameworks during the implementation process; and methods and approaches to implementation research. The content in our included capacity-building initiatives closely aligned with the first two pillars, with less emphasis on the third pillar of implementation research. Moore and Khan identified 37 competencies linked to nine core implementation activities: inspire stakeholders and develop relationships, build implementation teams, understand the problem, use evidence to inform all aspects of implementation, assess the context, facilitate implementation, evaluate, plan for sustainability, and brokering knowledge [23]. The capacity-building initiatives we examined in our study generally covered these nine activities, although some were described less frequently (e.g., building an implementation team, sustainability). While the depth of our data did not allow for a direct comparison between the capacity-building initiative content and the more detailed individual competencies, future work should explore the alignment between training content and current and emerging competencies for implementation practice and science. For instance, novel competencies are emerging related to equity considerations in implementation science [109]. While some teams in our study described including new content on equity in their capacity-building initiatives, further work is needed to explore how this training content aligns with these emerging competencies, how effectively it is developing implementation practitioners’ capacity to integrate equity considerations during implementation, and whether there are differences in equity considerations for implementation research versus implementation practice.

We identified several areas where, despite learning content in the capacity-building initiative, practitioners might experience challenges applying this knowledge in practice. First, although about 70% of the capacity-building initiatives in our study included content on how to find, appraise, and adapt evidence, there were concerns about whether learners could (or should) action these skills in day-to-day practice, given the time-intensive nature. This concern aligns with a systematic review that found “lack of time” as a top barrier to healthcare providers searching for, appraising, and learning from evidence [110]. Support from librarians has been shown to have positive outcomes (e.g., time savings for healthcare providers, more timely information for decision making) [111], although we acknowledge that librarians may not be easily accessible in all practice-based settings. Second, nearly all teams included content on monitoring and evaluation. However, based on the collective experiences of our team of implementation scientists and implementation practitioners, monitoring and evaluation are often not done (or not done in depth) in practice-based settings. Setting up effective data collection and monitoring systems has been identified as one of the top ten challenges to improving quality in healthcare, with settings often lacking the required expertise and infrastructure [112]. It is possible that the high proportion of teams including monitoring and evaluation content in their training is in response to this gap and an attempt to better equip learners with the required knowledge to effectively apply these skills in their settings.

The topic of sustainability was included by less than two-thirds of the teams. Given the growing attention on sustainability and scalability [113–115], this was surprising. There are several potential explanations. First, it is possible that sustainability concepts were integrated throughout the other content and not explicitly articulated as a separate content area by study participants. Second, most of the capacity-building initiatives in our study were time-limited, introductory courses. While most capacity-building initiatives introduced process models (e.g., KTA framework [36], Quality Implementation Framework [52], EPIS [58, 59]), which encourage consideration of the full implementation process from planning to sustainability, it is possible that the focus of the training was on the earlier phases of the models, with less attention to the longer-term activities of sustainability and scalability. However, sustainability needs to be considered early and often [116, 117] and it is worth considering who bears this responsibility. Johnson et al. [118] raised a similar question and recommended sustainability planning be a “dynamic, multifaceted approach with the involvement of all those who have a stake in sustainability such as funders, researchers, practitioners, and program beneficiaries” [118] ^(p. 7)^. It is thus important to ensure that capacity-building initiatives are equipping learners with the knowledge and skills to enhance sustainability and scalability throughout the full implementation process.

All teams described the importance of relational skills in the implementation process, from forming and maintaining a core implementation team, to engaging interested and affected parties in the implementation process, to effectively leading and facilitating implementation. Relational skills are required to work effectively in implementation practice, with about half of the 37 implementation core competencies being relational in nature [23]. In addition, an international survey of implementation experts most frequently identified collaboration knowledge and skills (e.g., interpersonal skills, networking and relationship building, teamwork and leadership skills, motivational skills, and ability to work with other disciplines and cultures) as the most helpful competency [24]. Our study also provided several examples of how this relational content is evolving in alignment with societal priorities and emerging areas in the fields of knowledge translation and implementation science, including integrated knowledge translation [28] and co-production [119] approaches, power differences and dynamics [120], equity, diversity, and inclusion, intersectionality considerations [121–126], and taking a principled approach to partnerships [127, 128]. It is promising that many teams offering capacity-building initiatives are staying abreast of these latest advances and priorities in developing the knowledge and skills of implementation practitioners.

### Strengths and limitations

We used a comprehensive recruitment approach to enroll a geographically diverse sample of participants with a variety of implementation, clinical, and research experiences, providing an international perspective on implementation practice training. We used a recent systematic review [7] as one strategy to identify published capacity-building initiatives; however, it is important to acknowledge that we did not conduct a comprehensive review of the literature and some capacity-building initiatives may have been missed. Furthermore, the inclusion of English-speaking participants only may have limited the identification and participation of other capacity-building initiative developers and deliverers. In addition, the current study focused on capacity-building initiatives offered primarily in the health sector. Implementation science and practice span many fields, offering an opportunity to replicate this study design to examine commonalities and unique content needs across different regions and contexts.

The use of primary and multiple data collection methods facilitated the collection of in-depth information on both what content is covered in the capacity-building initiatives as well as how and why this content is included. However, it is important to acknowledge that we only received capacity-building initiative materials from 11 of the 20 programs, which may have limited the comprehensiveness of the information on each initiative. In addition, the discussion guide asked participants about “critical content” and participants may therefore have only highlighted the “core” content in the time-limited interviews and focus groups. As such, while our findings provide an overview of what experts in the field identify as important training content, this likely is not reflective of every possible topic covered across capacity-building initiatives. Furthermore, while study participants shared what they included in their initiatives and why those content areas are important, this may not be representative of the optimal training content for all settings. Given the purpose of this study was not to assess the outcomes of the capacity-building initiatives, we cannot ascertain whether specific capacity-building initiative content is associated with better learner or health-system outcomes, which is an important area for future work.

Although this work extends our knowledge of key training content for implementation practice, content and curriculum are just one component of designing and delivering effective implementation practice training programs. Our team is currently working to synthesize additional data to describe the structure, format, and evaluation approaches of the capacity-building initiatives, as well as describe the experiences of the teams who facilitate the training.

## Conclusions

The results of this study highlight what experienced capacity-building initiative developers and deliverers identify as essential content for teaching implementation practice. These learnings may be informative to researchers, educators, and implementation practitioners working to develop, refine, and deliver capacity-building initiatives to enhance the translation of implementation science into practice. Future research is needed to better understand how the training content influences implementation outcomes.

### Supplementary Information

**Additional file 1.** GRAMMS reporting checklist.

**Additional file 2.** Survey and interview questions.

## Data Availability

The interview and focus group transcripts analyzed in this study are not publicly available due to them containing information that could compromise research participant privacy/consent. The questionnaire and data are available from the corresponding author on reasonable request.

## Abbreviation

TMFAs: Theories, models, frameworks, approaches

## References

[1] Morris ZS, Wooding S, Grant J (2011). The answer is 17 years, what is the question: understanding time lags in translational research. J R Soc Med.

[2] Westerlund A, Sundberg L, Nilsen P (2019). Implementation of implementation science knowledge: the research-practice gap paradox. Worldviews Evid Based Nurs.

[3] Rapport F, Smith J, Hutchinson K, Clay-Williams R, Churruca K, Bierbaum M (2022). Too much theory and not enough practice? The challenge of implementation science application in healthcare practice. J Eval Clin Pract.

[4] Beidas RS, Dorsey S, Lewis CC, Lyon AR, Powell BJ, Purtle J (2022). Promises and pitfalls in implementation science from the perspective of US-based researchers: learning from a pre-mortem. Implement Sci.

[5] Albers B, Metz A, Burke K (2020). Implementation support practitioners: a proposal for consolidating a diverse evidence base. BMC Health Serv Res.

[6] Straus SE, Sales A, Wensing M, Michie S, Kent B, Foy R (2015). Education and training for implementation science: our interest in manuscripts describing education and training materials. Implement Sci.

[7] Davis R, D’Lima D (2020). Building capacity in dissemination and implementation science: a systematic review of the academic literature on teaching and training initiatives. Implement Sci.

[8] Kislov R, Waterman H, Harvey G, Boaden R (2014). Rethinking capacity building for knowledge mobilisation: developing multilevel capabilities in healthcare organisations. Implement Sci.

[9] Proctor E, Chambers DA (2017). Training in dissemination and implementation research: a field-wide perspective. Transl Behav Med.

[10] Park JS, Moore JE, Sayal R, Holmes BJ, Scarrow G, Graham ID (2018). Evaluation of the “foundations in knowledge translation” training initiative: preparing end users to practice KT. Implement Sci.

[11] Moore JE, Rashid S, Park JS, Khan S, Straus SE (2018). Longitudinal evaluation of a course to build core competencies in implementation practice. Implement Sci.

[12] Bennett S, Whitehead M, Eames S, Fleming J, Low S, Caldwell E. Building capacity for knowledge translation in occupational therapy: learning through participatory action research. BMC Med Educ. 2–16;16(1):257. 10.1186/s12909-016-0771-5. 10.1186/s12909-016-0771-5 PMC5045617 27716230

[13] Eames S, Bennett S, Whitehead M, Fleming J, Low SO, Mickan S (2018). A pre-post evaluation of a knowledge translation capacity-building intervention. Aust Occup Ther J.

[14] Young AM, Cameron A, Meloncelli N, Barrimore SE, Campbell K, Wilkinson S (2023). Developing a knowledge translation program for health practitioners: allied health translating research into practice. Front Heal Serv.

[15] Provvidenza C, Townley A, Wincentak J, Peacocke S, Kingsnorth S (2020). Building knowledge translation competency in a community-based hospital: a practice-informed curriculum for healthcare providers, researchers, and leadership. Implement Sci.

[16] Black AT, Steinberg M, Chisholm AE, Coldwell K, Hoens AM, Koh JC (2021). Building capacity for implementation—the KT Challenge. Implement Sci Commun.

[17] Mosson R, Augustsson H, Bäck A, Åhström M, Von Thiele SU, Richter A (2019). Building implementation capacity (BIC): a longitudinal mixed methods evaluation of a team intervention. BMC Health Serv Res.

[18] Goodenough B, Fleming R, Young M, Burns K, Jones C, Forbes F (2017). Raising awareness of research evidence among health professionals delivering dementia care: are knowledge translation workshops useful?. Gerontol Geriatr Educ.

[19] Wyer PC, Umscheid CA, Wright S, Silva SA, Lang E (2015). Teaching Evidence Assimilation for Collaborative Health Care (TEACH) 2009–2014: building evidence-based capacity within health care provider organizations. EGEMS.

[20] Proctor E, Ramsey AT, Brown MT, Malone S, Hooley C, McKay V (2019). Training in Implementation Practice Leadership (TRIPLE): evaluation of a novel practice change strategy in behavioral health organizations. Implement Sci.

[21] U.S. Department of Veterans Affairs (2023). Implementation facilitation learning hub.

[22] Leppin AL, Baumann AA, Fernandez ME, Rudd BN, Stevens KR, Warner DO (2021). Teaching for implementation: a framework for building implementation research and practice capacity within the translational science workforce. J Clin Transl Sci.

[23] Moore JE, Khan S (2020). Core competencies for implementation practice.

[24] Schultes M-T, Aijaz M, Klug J, Fixsen DL (2021). Competences for implementation science: what trainees need to learn and where they learn it. Adv Health Sci Educ Theory Pract.

[25] O’Cathain A, Murphy E, Nicholl J (2008). The quality of mixed methods studies in health services research. J Heal Serv Res Policy.

[26] Fetters MD, Curry LA, Creswell JW (2013). Achieving integration in mixed methods designs-principles and practices. Health Serv Res.

[27] Sandelowski M (2000). Whatever happened to qualitative description?. Res Nurs Health.

[28] Kothari A, McCutcheon C, Graham ID (2017). Defining integrated knowledge translation and moving forward: a response to recent commentaries. Int J Heal Policy Manag.

[29] QSR International Pty Ltd. NVivo qualitative data analysis software, version 12 Pro. Burlington: QSR International Pty Ltd; 2017. Available from: https://support.qsrinternational.com/nvivo/s/.

[30] Hsieh HF, Shannon SE (2005). Three approaches to qualitative content analysis. Qual Health Res.

[31] Giesen L, Roeser A (2020). Structuring a team-based approach to coding qualitative data. Int J Qual Methods.

[32] The Center for Implementation (2023). TMFAs.

[33] Nilsen P (2015). Making sense of implementation theories, models and frameworks. Implement Sci.

[34] Harrison M, Graham ID (2021). Knowledge translation in nursing and healthcare: a roadmap to evidence-informed practice.

[35] Lincoln YS, Guba E (1985). Naturalistic inquiry.

[36] Graham ID, Logan J, Harrison MB, Straus SE, Tetroe J, Caswell W (2006). Lost in knowledge translation: time for a map?. J Contin Educ Health Prof.

[37] Atkins L, Francis J, Islam R, O’Connor D, Patey A, Ivers N (2017). A guide to using the theoretical domains framework of behaviour change to investigate implementation problems. Implement Sci.

[38] Michie S, van Stralen MM, West R (2011). The behaviour change wheel: a new method for characterising and designing behaviour change interventions. Implement Sci.

[39] Glasgow RE, Vogt TM, Boles SM (1999). Evaluating the public health impact of health promotion interventions: the RE-AIM framework. Am J Public Health.

[40] RE-AIM (2023). RE-AIM - Improving public health relevance and population health impact.

[41] Damschroder LJ, Reardon CM, Widerquist MAO, Lowery J (2022). The updated consolidated framework for implementation research based on user feedback. Implement Sci.

[42] CFIR Research Team-Center for Clinical Management Research (2023). Consolidated Framework for Implementation Research (CFIR).

[43] Chambers DA, Glasgow RE, Stange KC (2013). The dynamic sustainability framework: addressing the paradox of sustainment amid ongoing change. Implement Sci.

[44] Maher L, Gustafson D, Evans A (2010). Sustainability model and guide.

[45] Wandersman A, Duffy J, Flaspohler P, Noonan R, Lubell K, Stillman L (2008). Bridging the gap between prevention research and practice: the interactive systems framework for dissemination and implementation. Am J Community Psychol.

[46] Kitson AL, Rycroft-Malone J, Harvey G, Mccormack B, Seers K, Titchen A (2008). Evaluating the successful implementation of evidence into practice using the PARiHS framework: theoretical and practical challenges. Implement Sci.

[47] Wandersman A, Chien VH, Katz J (2012). Toward an evidence-based system for innovation support for implementing innovations with quality: tools, training, technical assistance, and quality assurance/quality improvement. Am J Community Psychol.

[48] Canadian Institutes of Health Research. Guide to knowledge translation planning at CIHR: integrated and end-of-grant approaches. Ottawa; 2012. Available from: https://cihr-irsc.gc.ca/e/documents/kt_lm_ktplan-en.pdf.

[49] May C, Finch T (2009). Implementing, embedding, and integrating practices: an outline of normalization process theory. Sociology.

[50] May C, Rapley T, Mair FS, Treweek S, Murray E, Ballini L (2015). Normalization process theory on-line users’ manual, toolkit and NoMAD instrument.

[51] Proctor E, Silmere H, Raghavan R, Hovmand P, Aarons G, Bunger A (2011). Outcomes for implementation research: conceptual distinctions, measurement challenges, and research agenda. Adm Policy Ment Health.

[52] Meyers DC, Durlak JA, Wandersman A (2012). The quality implementation framework: a synthesis of critical steps in the implementation process. Am J Community Psychol.

[53] Rogers EM (2003). Diffusion of innovations.

[54] Moore G, Campbell M, Copeland L, Craig P, Movsisyan A, Hoddinott P (2021). Adapting interventions to new contexts—the ADAPT guidance. BMJ.

[55] Pathman DE, Konrad TR, Freed GL, Freeman VA, Koch GG (1996). the awareness-to-adherence model of the steps to clinical guideline compliance. Med Care.

[56] Harrison MB, van den Hoek J, Graham ID (2014). CAN-IMPLEMENT: planning for best-practice implementation.

[57] Braithwaite J, Churruca K, Long JC, Ellis LA, Herkes J (2018). When complexity science meets implementation science: a theoretical and empirical analysis of systems change. BMC Med.

[58] Moullin JC, Dickson KS, Stadnick NA, Rabin B, Aarons GA (2019). Systematic review of the Exploration, Preparation, Implementation, Sustainment (EPIS) framework. Implement Sci.

[59] EPIS Framework. The EPIS implementation framework. Available from: https://episframework.com/. Cited 2023 Oct 16.

[60] Hawe P (2015). Lessons from complex interventions to improve health. Annu Rev Public Health.

[61] Stirman SW, Baumann AA, Miller CJ (2019). The FRAME: an expanded framework for reporting adaptations and modifications to evidence-based interventions. Implement Sci.

[62] Langley G, Moen R, Nolan K, Nolan T, Norman C, Provost L (2009). The improvement guide: a practical approach to enhancing organizational performance.

[63] Hilton K, Anderson A (2018). IHI psychology of change framework to advance and sustain improvement.

[64] Parker G, Kastner M, Born K, Berta W (2021). Development of an implementation process model: a Delphi study. BMC Health Serv Res..

[65] Buckwalter KC, Cullen L, Hanrahan K, Kleiber C, McCarthy AM, Iowa Model Collaborative (2017). Iowa model of evidence-based practice: revisions and validation. Worldviews Evid Based Nurs.

[66] Schiffer E (2007). Net-Map toolbox: influence mapping of social networks.

[67] Valente TW, Palinkas LA, Czaja S, Chu K-H, Brown CH (2015). Social network analysis for program implementation. PLoS ONE.

[68] Graham ID, Logan J (2004). Innovations in knowledge transfer and continuity of care. Can J Nurs Res.

[69] Domlyn AM, Scott V, Livet M, Lamont A, Watson A, Kenworthy T (2021). R = MC2 readiness building process: a practical approach to support implementation in local, state, and national settings. J Community Psychol.

[70] Bausch KC (2001). Soft systems theory. The emerging consensus in social systems theory.

[71] Khalil H, Lakhani A (2022). Using systems thinking methodologies to address health care complexities and evidence implementation. JBI Evid Implement.

[72] Flaspohler PD, Meehan C, Maras MA, Keller KE (2012). Ready, willing, and able: developing a support system to promote implementation of school-based prevention programs. Am J Community Psychol.

[73] Francis J, Presseau J, Llewellyn C, Ayers S, McManus C (2019). Healthcare practitioner behaviour. Cambridge Handb Psychol Heal Med.

[74] Ajzen I (1991). The theory of planned behavior. Organ Behav Hum Decis Process.

[75] University of Colorado Denver (2023). Dissemination & implementation models in health.

[76] North Carolina Translational and Clinical Sciences Institute (2023). Theory, Model, and Framework Comparison and Selection Tool (T-CaST).

[77] Birken SA, Rohweder CL, Powell BJ, Shea CM, Scott J, Leeman J (2018). T-CaST: an implementation theory comparison and selection tool. Implement Sci.

[78] Powell BJ, Waltz TJ, Chinman MJ, Damschroder LJ, Smith JL, Matthieu MM (2015). A refined compilation of implementation strategies: results from the Expert Recommendations for Implementing Change (ERIC) project. Implement Sci.

[79] Michie S, Richardson M, Johnston M, Abraham C, Francis J, Hardeman W (2013). The behavior change technique taxonomy (v1) of 93 hierarchically clustered techniques: building an international consensus for the reporting of behavior change interventions. Ann Behav Med.

[80] Knowledge Translation Program (2016). Practicing knowledge translation: implementing evidence.

[81] CADTH. CADTH Rx for change database. Available from: https://cadth-login.wicketcloud.com/login?service=https%3A//www.cadth.ca/casservice%3Fdestination%3D/user/login%253Fdestination%253D%25252Frx-change. Cited 2023 Apr 26.

[82] Centre for Healthcare Innovation. Methods of patient & public engagement: a guide. Winnipeg; 2020. Available from: https://umanitoba.ca/centre-for-healthcare-innovation/sites/centre-for-healthcare-innovation/files/2021-11/methods-of-patient-and-public-engagement-guide.pdf.

[83] Bowen S. A guide to evaluation in health research. Ottawa; 2013. Available from: https://cihr-irsc.gc.ca/e/documents/kt_lm_guide_evhr-en.pdf.

[84] Pipes T. Work more effectively and productively with the Eisenhower Matrix. Medium. 2017. Available from: https://medium.com/taking-note/work-more-effectively-and-productively-with-the-eisenhower-matrix-998091a14b3a. Cited 2023 Apr 26.

[85] Effective Practice and Organisation of Care (EPOC). EPOC Taxonomy. 2015. Available from: epoc.cochrane.org/epoc-taxonomy.

[86] RAND Corporation (2023). Learn and use getting to outcomes®.

[87] Health Consumers Queensland. Health Consumers Queensland. Available from: https://www.hcq.org.au/. Cited 2023 Apr 26.

[88] International Association for Public Participation (IAP2). International Association for Public Participation (IAP2). Available from: https://www.iap2.org/mpage/Home. Cited 2023 Apr 26.

[89] Hailey D, Grimshaw J, Eccles M, Mitton C, Adair CE, McKenzie E, et al. Effective dissemination of findings from research. Alberta; 2008. Available from: https://www.ihe.ca/publications/effective-dissemination-of-findings-from-research-a-compilation-of-essays.

[90] Fernandez ME, ten Hoor GA, van Lieshout S, Rodriguez SA, Beidas RS, Parcel G (2019). Implementation mapping: using intervention mapping to develop implementation strategies. Front Public Heal.

[91] King’s Improvement Science. KIS guide to evaluation resources. London; 2018. Available from: https://kingsimprovementscience.org/cms-data/resources/KIS_evaluation_guide_December_2018.pdf.

[92] Barwick MA. Knowledge translation planning template. Toronto; 2008. Available from: https://www.sickkids.ca/en/learning/continuing-professional-development/knowledge-translation-training/knowledge-translation-planning-template-form/.

[93] KTDRR (Center on Knowledge Translation for Disability & Rehabilitation Research) (2023). KT strategies database.

[94] NHS Institute for Innovation and Improvement (2010). Sustainability guide.

[95] National Implementation Research Network (NIRN). NIRN. Available from: https://nirn.fpg.unc.edu/. Cited 2023 Apr 26.

[96] Ready, Set, Change! A readiness for change decision support tool. Available from: http://readiness.knowledgetranslation.ca/. Cited 2023 Apr 26. 10.1186/s12911-016-0262-y PMC4765048 26907792

[97] Institute for Healthcare Improvement. SBAR Tool: Situation-Background-Assessment-Recommendation | IHI - Institute for Healthcare Improvement. 2023. Available from: https://www.ihi.org/resources/Pages/Tools/SBARToolkit.aspx. Cited 2023 Apr 26.

[98] Washington University in St. Louis (2023). Program Sustainability Assessment Tool (PSAT) and Clinical Sustainability Assessment Tool (CSAT).

[99] Brown CH, Curran G, Palinkas LA, Aarons GA, Wells KB, Jones L (2017). An overview of research and evaluation designs for dissemination and implementation. Annu Rev Public Health.

[100] The Community Engagement Network. The engagement toolkit. Melbourne; 2005. Available from: https://www.betterevaluation.org/tools-resources/engagement-toolkit

[101] The Theory and Techniques Tool. Available from: https://theoryandtechniquetool.humanbehaviourchange.org/tool. Cited 2023 Apr 26.

[102] Flottorp SA, Oxman AD, Krause J, Musila NR, Wensing M, Godycki-Cwirko M (2013). A checklist for identifying determinants of practice: a systematic review and synthesis of frameworks and taxonomies of factors that prevent or enable improvements in healthcare professional practice. Implement Sci.

[103] Lynch EA, Mudge A, Knowles S, Kitson AL, Hunter SC, Harvey G (2018). “There is nothing so practical as a good theory”: a pragmatic guide for selecting theoretical approaches for implementation projects. BMC Health Serv.

[104] Bhuiya AR, Sutherland J, Boateng R, Bain T, Skidmore B, Perrier L, et al. A scoping review reveals candidate quality indicators of knowledge translation and implementation science practice tools. J Clin Epidemiol. 2023;S0895-4356(23):00281-0. 10.1016/j.jclinepi.2023.10.021. 10.1016/j.jclinepi.2023.10.021 37939744

[105] Strifler L, Cardoso R, McGowan J, Cogo E, Nincic V, Khan PA (2018). Scoping review identifies significant number of knowledge translation theories, models, and frameworks with limited use. J Clin Epidemiol.

[106] Strifler L, Barnsley JM, Hillmer M, Straus SE (2020). Identifying and selecting implementation theories, models and frameworks: a qualitative study to inform the development of a decision support tool. BMC Med Inform Decis Mak.

[107] Barrimore SE, Cameron AE, Young AM, Hickman IJ, Campbell KL (2020). Translating research into practice: how confident are allied health clinicians?. J Allied Health.

[108] Metz A, Louison L, Burke K, Albers B, Ward C. Implementation support practitioner profile: guiding principles and core competencies for implementation practice. Chapel Hill; 2020. Available from: https://nirn.fpg.unc.edu/sites/nirn.fpg.unc.edu/files/resources/IS%20Practice%20Profile-single%20page%20printing-v10-October%202022.pdf.

[109] Huebschmann AG, Johnston S, Davis R, Kwan BM, Geng E, Haire-Joshu D (2022). Promoting rigor and sustainment in implementation science capacity building programs: a multi-method study. Implement Res Pract.

[110] Sadeghi-Bazargani H, Tabrizi JS, Azami-Aghdash S (2014). Barriers to evidence-based medicine: a systematic review. J Eval Clin Pract.

[111] Perrier L, Farrell A, Ayala AP, Lightfoot D, Kenny T, Aaronson E (2014). Effects of librarian-provided services in healthcare settings: a systematic review. J Am Med Informatics Assoc.

[112] Dixon-Woods M, McNicol S, Martin G (2012). Ten challenges in improving quality in healthcare: lessons from the Health Foundation’s programme evaluations and relevant literature. BMJ Qual Saf.

[113] Straus SE, Rapport F, Clay-Williams R, Braithwaite J (2022). Implementation sustainability. Implement Sci Key Concepts.

[114] Corôa RDC, Gogovor A, Ben Charif A, Hassine A Ben, Zomahoun HTV, McLean RKD, et al. Evidence on scaling in health and social care: an umbrella review. Milbank Q. 2023. 10.1111/1468-0009.12649. 10.1111/1468-0009.12649 PMC10509507 37186312

[115] Moore JE, Mascarenhas A, Bain J, Straus SE (2017). Developing a comprehensive definition of sustainability. Implement Sci.

[116] Moore JE (2023). Sustainability: What is it? Why is it important? How are readiness, context, and sustainability related?.

[117] Kwan BM, Brownson RC, Glasgow RE, Morrato EH, Luke DA (2022). Designing for dissemination and sustainability to promote equitable impacts on health. Annu Rev Public Health.

[118] Johnson AM, Moore JE, Chambers DA, Rup J, Dinyarian C, Straus SE (2019). How do researchers conceptualize and plan for the sustainability of their NIH R01 implementation projects?. Implement Sci.

[119] Graham ID, Rycroft-Malone J, Kothari A, McCutcheon C, editors. Research coproduction in healthcare. Hoboken: Wiley; 2022.

[120] Finley EP, Closser S, Sarker M, Hamilton AB (2023). Editorial: the theory and pragmatics of power and relationships in implementation. Front Heal Serv.

[121] Presseau J, Kasperavicius D, Rodrigues IB, Braimoh J, Chambers A, Etherington C (2022). Selecting implementation models, theories, and frameworks in which to integrate intersectional approaches. BMC Med Res Methodol.

[122] Etherington C, Rodrigues IB, Giangregorio L, Graham ID, Hoens AM, Kasperavicius D (2020). Applying an intersectionality lens to the theoretical domains framework: a tool for thinking about how intersecting social identities and structures of power influence behaviour. BMC Med Res Methodol.

[123] Sibley KM, Kasperavicius D, Rodrigues IB, Giangregorio L, Gibbs JC, Graham ID (2022). Development and usability testing of tools to facilitate incorporating intersectionality in knowledge translation. BMC Health Serv Res.

[124] Tannenbaum C, Greaves L, Graham ID (2016). Why sex and gender matter in implementation research. BMC Med Res Methodol.

[125] Brownson RC, Kumanyika SK, Kreuter MW, Haire-Joshu D (2021). Implementation science should give higher priority to health equity. Implement Sci.

[126] Fort MP, Manson SM, Glasgow RE (2023). Applying an equity lens to assess context and implementation in public health and health services research and practice using the PRISM framework. Front Heal Serv.

[127] Gainforth HL, Hoekstra F, McKay R, McBride CB, Sweet SN, Martin Ginis KA (2021). Integrated knowledge translation guiding principles for conducting and disseminating spinal cord injury research in partnership. Arch Phys Med Rehabil.

[128] Hoekstra F, Trigo F, Sibley KM, Graham ID, Kennefick M, Mrklas KJ (2023). Systematic overviews of partnership principles and strategies identified from health research about spinal cord injury and related health conditions: a scoping review. J Spinal Cord Med.

