# Supplementary material for: Essential content for teaching implementation practice in healthcare: a mixed-methods study of teams offering capacity-building initiatives
Source: Implement Sci Commun. 2023 Nov 27;4:151. doi: 10.1186/s43058-023-00525-0 (PMC10680357; doi:10.1186/s43058-023-00525-0)
Supplement: Supplementary file 1 — Additional file 1. GRAMMS reporting checklist. [file 43058_2023_525_MOESM1_ESM.docx]

**Additional File 1: Completed reporting checklist**

*This is a supplemental file to a full manuscript published in Implementation Science Communications. For full copyright and citation information see doi: 10.1186/s43058-023-00525-0*

**Good Reporting of A Mixed Methods Study (GRAMMS)**

| **Item**  **#** | **GRAMMS item description** | **Manuscript Section** | **Page #** |
| --- | --- | --- | --- |
| 1 | Describe the justification for using a mixed methods approach to the research question. | Methods - Integration | 11 |
| 2 | Describe the design in terms of the purpose, priority and sequence of methods. | Methods - Design  Methods - Data collection  Methods - Integration | 7  7-8  9 |
| 3 | Describe each method in terms of sampling, data collection and analysis. | Methods - Study participants  Methods - Data collection Methods - Data analysis | 6-7  8-10  10-11 |
| 4 | Describe where integration has occurred, how it has occurred and who has participated in it. | Methods - Integration | 11 |
| 5 | Describe any limitation of one method associated with the presence of the other method. | N/A | — |
| 6 | Describe any insights gained from mixing or integrating methods | Methods - Integration  Discussion - Strengths & limitations | 11  27-28 |

*O'Cathain A, Murphy E, Nicholl J. The quality of mixed methods studies in health services research. J Health Serv Res Policy. 2008;13: 92-98.*
