# Supplementary material for: Essential content for teaching implementation practice in healthcare: a mixed-methods study of teams offering capacity-building initiatives
Source: Implement Sci Commun. 2023 Nov 27;4:151. doi: 10.1186/s43058-023-00525-0 (PMC10680357; doi:10.1186/s43058-023-00525-0)
Supplement: Supplementary file 2 — Additional file 2. Survey and interview questions. [file 43058_2023_525_MOESM2_ESM.docx]

**Additional File 2: Questions from questionnaire and discussion guide analyzed in this study**

*This is a supplemental file to a full manuscript published in Implementation Science Communications. For full copyright and citation information see doi: 10.1186/s43058-023-00525-0*

| **SURVEY QUESTIONS**  *(used in this analysis)* | |
| --- | --- |
|  | |
| What are the stated aims or goals of your capacity building initiative? |  |
|  |  |
| What implementation science theories, models and frameworks are included in your capacity building initiative? |  |
|  |  |
| Is there a particular theoretical approach underpinning your capacity building initiative (e.g., pedagogical theory, implementation theory)? | Yes  No  If “yes”, please specify: |
|  |  |
| What practical implementation skills do you teach in your capacity building initiative? |  |
|  |  |
| Is relationship/team building part of your course content/curriculum? | Yes  No  If “yes”, please explain what aspects: |
|  |  |
| Does your capacity building initiative include information on how to engage health consumers and patients in the implementation process? | Yes  No |
|  | |
| If you have any additional comments on your capacity building initiative, please enter them here. |  |

| **INTERVIEW QUESTIONS**  (used in this analysis) |
| --- |

1. How did you ensure that your capacity building initiative met the needs of the practitioners? Did any knowledge users take part in the development?
   - Tell me about your approach to working with stakeholders (and patients, if applicable)
2. I have looked over the survey responses [*provided by you or your team*] and want to talk more in depth about your capacity building initiative.
   - Tell me more about the **content/curriculum** in your capacity building initiative *[probe specific survey questions from the “content & curriculum” section as needed, plus additional probes below].*
     - What is critical content? Was all of it essential or were some parts more important than others and how come?
     - Tell me about the relationship and team building content in your capacity building initiative (if applicable).
     - Do you change the content depending on the audience? How and why?
3. Now let’s talk about what you have learned related to how to best teach practitioners implementation.
   - What have you learned about teaching others about how to implement best practices?
   - What concepts/skills do participants finding challenging to acquire? What strategies did you use to help participants with these challenges?
   - Many current capacity building initiatives are designed for implementation *scientists*. Are there any key differences in the content and format of your implementation *practice* capacity building initiative, compared to a one targeted at scientists? Any key similarities?
4. Overall, what worked well with the capacity-building initiative?
5. What challenges have you encountered delivering the capacity-building initiative?
   - Has anything gone wrong?
   - Anything to avoid?
6. Thinking about these factors that worked well and those that were more challenging, if you could re-design the capacity building initiative:
   - What would you change? How come?
   - What aspects would you keep? How come?
7. Do you have any other experiences, observations or insights about developing and/or delivering capacity building initiatives that you would like to share with me today?
